# Notices

**Published:** 1867-09

**Authors:** 


### NOTICES.

"TIMES of Knox and Mary Queen of Scots" is a beautifully bound volume in crimson cloth, of 359 pages, by Mrs. S. T. Martyn, author of "The Women of the Bible," "Allen Cameron," etc., published by the American Tract Society, 150 Nassau Street, New York. It will be read with deep interest as a record of troublous times, giving as it does a truthful view, religious and historical, of an eventful age. Also, "The Bible Reader's Help," from the Religious Tract Society of London, revised and enlarged, well intended for that large class of Bible readers who have not access to costlier works illustrating Bible meaning. The Society have wisely issued other Bible Helps, as "The Family Bible with Notes;" "The Illustrated Bible Dictionary." "Bible Atlas and Bible Test Book." Also, "Toils and Triumphs of Union Missionary Colporter."

The Messrs. Broughton and Wyman, 13 Bible House, New York, have published "Lottie Wild's Picnic" in beautiful style. It is founded on fact, and dedicated to the First Congregational Society in Marblehead. 198 pages, purple cloth.

THE SOUTHERN PRESBYTERIAN REVIEW, $3 a year, conducted by an Association of Ministers in Columbia, South Carolina. Its mechanical execution, thick white paper and large clear type, would do credit to any Northern press, while the subjects treated in this 17th volume show its sterling character. Among the subjects discussed are, Buckle's History af Civilization; The Life and Times of Dr. Spring, of New York City; Infant Baptism; Study of Language as a Training of the Mind, —a most suggestive article; Death, the Resurrection and the Intermediate State; Renan's Origin of Christianity; Science of Pastoral Theology; Modern Infidelity; Pastoral Relations and Duties; Female Education, &c., &c. This excellent Review ought to secure the patronage of Northern christians, and we trust it will.

THE ENGLISH EXILE; or William Tyndall at Home and Abroad, by Mrs. S. T. Martyn, is the title of a useful, interesting and instructive volume of The American Tract Society, at 150 Nassau Street, New York.

TRAVELLERS, ATTENTION! The APPLETONS, 445 Broadway, New York, and Trubner & Co., Paternoster Row, London, have published a "Hand-Book of American Travel," The Northern Tour, with valuable maps of the leading routs of travel and of the principal cities, by Edward H. Hall, being the ninth annual edition, including New Mexico and the Dominion of Canada, with a copious and convenient Index. Not among the least of its practicalities is the announcement of the principal Hotels throughout the country, Tables of Distances across the continent, &c., &c. The book gives a succinct account of almost every place of any note north of Mason and Dixon's Line.

FATHERS AND SONS, a Russian novel, translated by Eugene Schuyler, published by Leypoldt & Holt in an attractive volume of 248 pages. It was published originally in a Moscow Review and attracted considerable attention in both political and social circles. It gives a truthful and interesting insight into Russian life, literature and institutions, and as such, is of greater value, perhaps, than any similar Russian work published in this country.

The same house has published several seasonable books or critical and social essays, reprinted from the NEW YORK NATION, 12mo, cloth, $1.50. The Man with a Broken Ear, by Edmund About,—$1.50. The Hugenot Galley Slave. The Journal of Maurice de Guisin. Thackeray's Works, uniform edition Charles Kingsley's Works, uniform edition, 16mo, from $1.25 to $2.00 per volume. Romance of a Poor Young Man. Human Follies, &c. &c.

